# Why Most Published Research Findings Are False

**DOI:** 10.1371/journal.pmed.0020124

**Published:** 2005-08-30

**Authors:** John P. A. Ioannidis

## Abstract

There is increasing concern that most current published research findings are false. The probability that a research claim is true may depend on study power and bias, the number of other studies on the same question, and, importantly, the ratio of true to no relationships among the relationships probed in each scientific field. In this framework, a research finding is less likely to be true when the studies conducted in a field are smaller; when effect sizes are smaller; when there is a greater number and lesser preselection of tested relationships; where there is greater flexibility in designs, definitions, outcomes, and analytical modes; when there is greater financial and other interest and prejudice; and when more teams are involved in a scientific field in chase of statistical significance. Simulations show that for most study designs and settings, it is more likely for a research claim to be false than true. Moreover, for many current scientific fields, claimed research findings may often be simply accurate measures of the prevailing bias. In this essay, I discuss the implications of these problems for the conduct and interpretation of research.

## Abstract 2

Published research findings are sometimes refuted by subsequent evidence, says Ioannidis, with ensuing confusion and disappointment.

Published research findings are sometimes refuted by subsequent evidence, with ensuing confusion and disappointment. Refutation and controversy is seen across the range of research designs, from clinical trials and traditional epidemiological studies [1–3] to the most modern molecular research [4,5]. There is increasing concern that in modern research, false findings may be the majority or even the vast majority of published research claims [6–8]. However, this should not be surprising. It can be proven that most claimed research findings are false. Here I will examine the key factors that influence this problem and some corollaries thereof.

## Modeling the Framework for False Positive Findings

Several methodologists have pointed out [9–11] that the high rate of nonreplication (lack of confirmation) of research discoveries is a consequence of the convenient, yet ill-founded strategy of claiming conclusive research findings solely on the basis of a single study assessed by formal statistical significance, typically for a *p*-value less than 0.05. Research is not most appropriately represented and summarized by *p*-values, but, unfortunately, there is a widespread notion that medical research articles should be interpreted based only on *p*-values. Research findings are defined here as any relationship reaching formal statistical significance, e.g., effective interventions, informative predictors, risk factors, or associations. “Negative” research is also very useful. “Negative” is actually a misnomer, and the misinterpretation is widespread. However, here we will target relationships that investigators claim exist, rather than null findings.

It can be proven that most claimed research findings are false

As has been shown previously, the probability that a research finding is indeed true depends on the prior probability of it being true (before doing the study), the statistical power of the study, and the level of statistical significance [10,11]. Consider a 2 × 2 table in which research findings are compared against the gold standard of true relationships in a scientific field. In a research field both true and false hypotheses can be made about the presence of relationships. Let *R* be the ratio of the number of “true relationships” to “no relationships” among those tested in the field. *R* is characteristic of the field and can vary a lot depending on whether the field targets highly likely relationships or searches for only one or a few true relationships among thousands and millions of hypotheses that may be postulated. Let us also consider, for computational simplicity, circumscribed fields where either there is only one true relationship (among many that can be hypothesized) or the power is similar to find any of the several existing true relationships. The pre-study probability of a relationship being true is *R*/(*R* + 1). The probability of a study finding a true relationship reflects the power 1 - β (one minus the Type II error rate). The probability of claiming a relationship when none truly exists reflects the Type I error rate, α. Assuming that *c* relationships are being probed in the field, the expected values of the 2 × 2 table are given in Table 1. After a research finding has been claimed based on achieving formal statistical significance, the post-study probability that it is true is the positive predictive value, PPV. The PPV is also the complementary probability of what Wacholder et al. have called the false positive report probability [10]. According to the 2 × 2 table, one gets PPV = (1 - β)*R*/(*R* - βR + α). A research finding is thus more likely true than false if (1 - β)*R* > α. Since usually the vast majority of investigators depend on a = 0.05, this means that a research finding is more likely true than false if (1 - β)*R* > 0.05.

**Table 1:**
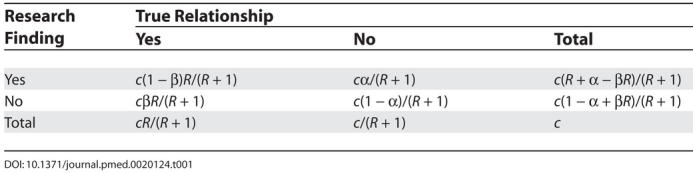
Research Findings and True Relationships

What is less well appreciated is that bias and the extent of repeated independent testing by different teams of investigators around the globe may further distort this picture and may lead to even smaller probabilities of the research findings being indeed true. We will try to model these two factors in the context of similar 2 × 2 tables.

## Bias

First, let us define bias as the combination of various design, data, analysis, and presentation factors that tend to produce research findings when they should not be produced. Let *u* be the proportion of probed analyses that would not have been “research findings,” but nevertheless end up presented and reported as such, because of bias. Bias should not be confused with chance variability that causes some findings to be false by chance even though the study design, data, analysis, and presentation are perfect. Bias can entail manipulation in the analysis or reporting of findings. Selective or distorted reporting is a typical form of such bias. We may assume that *u* does not depend on whether a true relationship exists or not. This is not an unreasonable assumption, since typically it is impossible to know which relationships are indeed true. In the presence of bias (Table 2), one gets PPV = ([1 - β]*R* + *u*β*R*)/(*R* + α − β*R* + *u* − *u*α + *u*β*R*), and PPV decreases with increasing *u*, unless 1 − β ≤ α, i.e., 1 − β ≤ 0.05 for most situations. Thus, with increasing bias, the chances that a research finding is true diminish considerably. This is shown for different levels of power and for different pre-study odds in Figure 1. Conversely, true research findings may occasionally be annulled because of reverse bias. For example, with large measurement errors relationships are lost in noise [12], or investigators use data inefficiently or fail to notice statistically significant relationships, or there may be conflicts of interest that tend to “bury” significant findings [13]. There is no good large-scale empirical evidence on how frequently such reverse bias may occur across diverse research fields. However, it is probably fair to say that reverse bias is not as common. Moreover measurement errors and inefficient use of data are probably becoming less frequent problems, since measurement error has decreased with technological advances in the molecular era and investigators are becoming increasingly sophisticated about their data. Regardless, reverse bias may be modeled in the same way as bias above. Also reverse bias should not be confused with chance variability that may lead to missing a true relationship because of chance.

**Figure 1:**
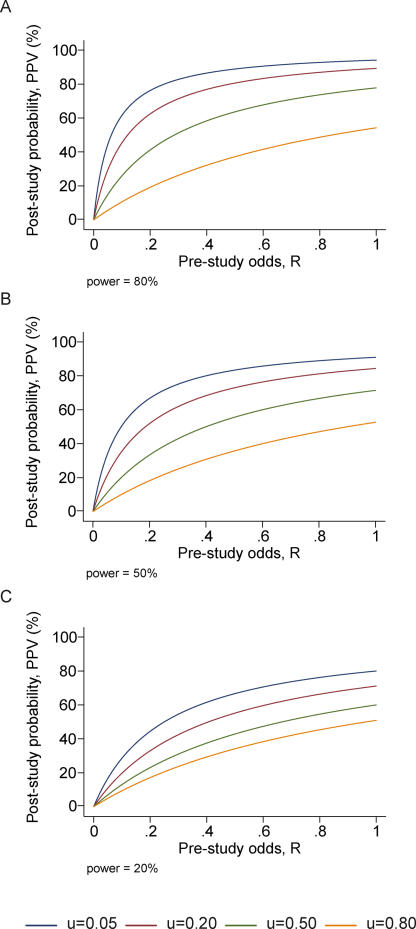
PPV (Probability That a Research Finding Is True) as a Function of the Pre-Study Odds for Various Levels of Bias, *u* Panels correspond to power of 0.20, 0.50, and 0.80.

**Table 2:**
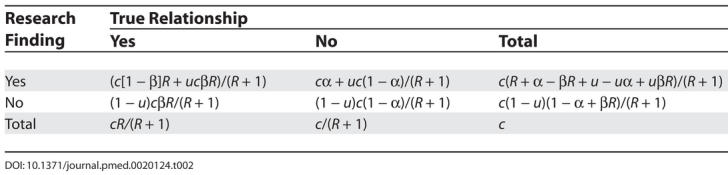
Research Findings and True Relationships in the Presence of Bias

## Testing by Several Independent Teams

Several independent teams may be addressing the same sets of research questions. As research efforts are globalized, it is practically the rule that several research teams, often dozens of them, may probe the same or similar questions. Unfortunately, in some areas, the prevailing mentality until now has been to focus on isolated discoveries by single teams and interpret research experiments in isolation. An increasing number of questions have at least one study claiming a research finding, and this receives unilateral attention. The probability that at least one study, among several done on the same question, claims a statistically significant research finding is easy to estimate. For *n* independent studies of equal power, the 2 × 2 table is shown in Table 3: PPV = *R*(1 − β^*n*^)/(*R* + 1 − [1 − α]^*n*^ − *R*β^*n*^) (not considering bias). With increasing number of independent studies, PPV tends to decrease, unless 1 - β < a, i.e., typically 1 − β < 0.05. This is shown for different levels of power and for different pre-study odds in Figure 2. For *n* studies of different power, the term β^*n*^ is replaced by the product of the terms β_*i*_ for *i* = 1 to *n*, but inferences are similar.

**Figure 2:**
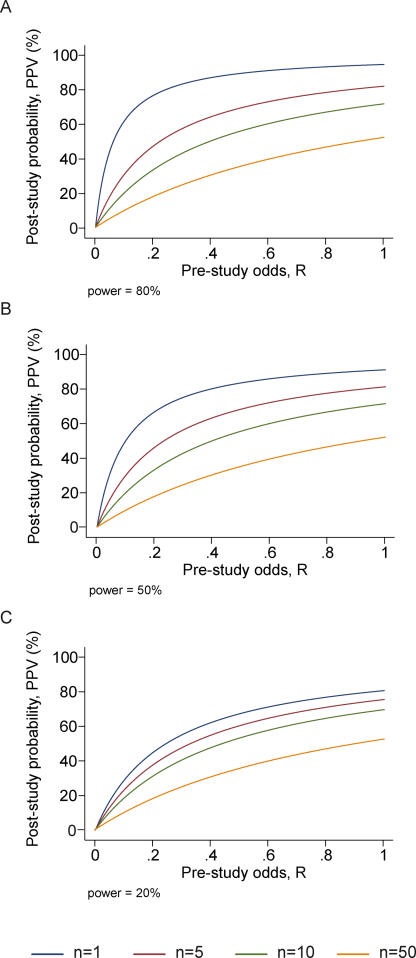
PPV (Probability That a Research Finding Is True) as a Function of the Pre-Study Odds for Various Numbers of Conducted Studies, *n* Panels correspond to power of 0.20, 0.50, and 0.80.

**Table 3:**
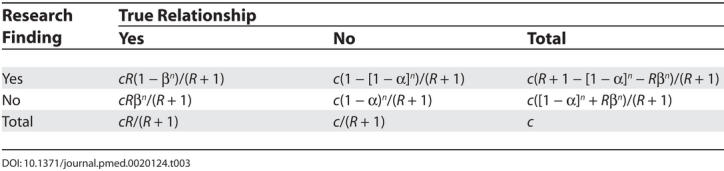
Research Findings and True Relationships in the Presence of Multiple Studies

## Corollaries

A practical example is shown in Box 1. Based on the above considerations, one may deduce several interesting corollaries about the probability that a research finding is indeed true.

**Box 1. An Example: Science at Low Pre-Study Odds**

Let us assume that a team of investigators performs a whole genome association study to test whether any of 100,000 gene polymorphisms are associated with susceptibility to schizophrenia. Based on what we know about the extent of heritability of the disease, it is reasonable to expect that probably around ten gene polymorphisms among those tested would be truly associated with schizophrenia, with relatively similar odds ratios around 1.3 for the ten or so polymorphisms and with a fairly similar power to identify any of them. Then *R* = 10/100,000 = 10^−4^, and the pre-study probability for any polymorphism to be associated with schizophrenia is also *R*/(*R* + 1) = 10^−4^. Let us also suppose that the study has 60% power to find an association with an odds ratio of 1.3 at α = 0.05. Then it can be estimated that if a statistically significant association is found with the *p*-value barely crossing the 0.05 threshold, the post-study probability that this is true increases about 12-fold compared with the pre-study probability, but it is still only 12 × 10^−4^.

Now let us suppose that the investigators manipulate their design, analyses, and reporting so as to make more relationships cross the *p* = 0.05 threshold even though this would not have been crossed with a perfectly adhered to design and analysis and with perfect comprehensive reporting of the results, strictly according to the original study plan. Such manipulation could be done, for example, with serendipitous inclusion or exclusion of certain patients or controls, post hoc subgroup analyses, investigation of genetic contrasts that were not originally specified, changes in the disease or control definitions, and various combinations of selective or distorted reporting of the results. Commercially available “data mining” packages actually are proud of their ability to yield statistically significant results through data dredging. In the presence of bias with *u* = 0.10, the post-study probability that a research finding is true is only 4.4 × 10^−4^. Furthermore, even in the absence of any bias, when ten independent research teams perform similar experiments around the world, if one of them finds a formally statistically significant association, the probability that the research finding is true is only 1.5 × 10^−4^, hardly any higher than the probability we had before any of this extensive research was undertaken!

**Corollary 1: The smaller the studies conducted in a scientific field, the less likely the research findings are to be true.** Small sample size means smaller power and, for all functions above, the PPV for a true research finding decreases as power decreases towards 1 − β = 0.05. Thus, other factors being equal, research findings are more likely true in scientific fields that undertake large studies, such as randomized controlled trials in cardiology (several thousand subjects randomized) [14] than in scientific fields with small studies, such as most research of molecular predictors (sample sizes 100-fold smaller) [15].

**Corollary 2: The smaller the effect sizes in a scientific field, the less likely the research findings are to be true.** Power is also related to the effect size. Thus research findings are more likely true in scientific fields with large effects, such as the impact of smoking on cancer or cardiovascular disease (relative risks 3–20), than in scientific fields where postulated effects are small, such as genetic risk factors for multigenetic diseases (relative risks 1.1–1.5) [7]. Modern epidemiology is increasingly obliged to target smaller effect sizes [16]. Consequently, the proportion of true research findings is expected to decrease. In the same line of thinking, if the true effect sizes are very small in a scientific field, this field is likely to be plagued by almost ubiquitous false positive claims. For example, if the majority of true genetic or nutritional determinants of complex diseases confer relative risks less than 1.05, genetic or nutritional epidemiology would be largely utopian endeavors.

**Corollary 3: The greater the number and the lesser the selection of tested relationships in a scientific field, the less likely the research findings are to be true.** As shown above, the post-study probability that a finding is true (PPV) depends a lot on the pre-study odds *(R)*. Thus, research findings are more likely true in confirmatory designs, such as large phase III randomized controlled trials, or meta-analyses thereof, than in hypothesis-generating experiments. Fields considered highly informative and creative given the wealth of the assembled and tested information, such as microarrays and other high-throughput discovery-oriented research [4,8,17], should have extremely low PPV.

**Corollary 4: The greater the flexibility in designs, definitions, outcomes, and analytical modes in a scientific field, the less likely the research findings are to be true.** Flexibility increases the potential for transforming what would be “negative” results into “positive” results, i.e., bias, *u*. For several research designs, e.g., randomized controlled trials [18–20] or meta-analyses [21,22], there have been efforts to standardize their conduct and reporting. Adherence to common standards is likely to increase the proportion of true findings. The same applies to outcomes. True findings may be more common when outcomes are unequivocal and universally agreed (e.g., death) rather than when multifarious outcomes are devised (e.g., scales for schizophrenia outcomes) [23]. Similarly, fields that use commonly agreed, stereotyped analytical methods (e.g., Kaplan-Meier plots and the log-rank test) [24] may yield a larger proportion of true findings than fields where analytical methods are still under experimentation (e.g., artificial intelligence methods) and only “best” results are reported. Regardless, even in the most stringent research designs, bias seems to be a major problem. For example, there is strong evidence that selective outcome reporting, with manipulation of the outcomes and analyses reported, is a common problem even for randomized trails [25]. Simply abolishing selective publication would not make this problem go away.

**Corollary 5: The greater the financial and other interests and prejudices in a scientific field, the less likely the research findings are to be true.** Conflicts of interest and prejudice may increase bias, *u*. Conflicts of interest are very common in biomedical research [26], and typically they are inadequately and sparsely reported [26,27]. Prejudice may not necessarily have financial roots. Scientists in a given field may be prejudiced purely because of their belief in a scientific theory or commitment to their own findings. Many otherwise seemingly independent, university-based studies may be conducted for no other reason than to give physicians and researchers qualifications for promotion or tenure. Such nonfinancial conflicts may also lead to distorted reported results and interpretations. Prestigious investigators may suppress via the peer review process the appearance and dissemination of findings that refute their findings, thus condemning their field to perpetuate false dogma. Empirical evidence on expert opinion shows that it is extremely unreliable [28].

**Corollary 6: The hotter a scientific field (with more scientific teams involved), the less likely the research findings are to be true.** This seemingly paradoxical corollary follows because, as stated above, the PPV of isolated findings decreases when many teams of investigators are involved in the same field. This may explain why we occasionally see major excitement followed rapidly by severe disappointments in fields that draw wide attention. With many teams working on the same field and with massive experimental data being produced, timing is of the essence in beating competition. Thus, each team may prioritize on pursuing and disseminating its most impressive “positive” results. “Negative” results may become attractive for dissemination only if some other team has found a “positive” association on the same question. In that case, it may be attractive to refute a claim made in some prestigious journal. The term Proteus phenomenon has been coined to describe this phenomenon of rapidly alternating extreme research claims and extremely opposite refutations [29]. Empirical evidence suggests that this sequence of extreme opposites is very common in molecular genetics [29].

These corollaries consider each factor separately, but these factors often influence each other. For example, investigators working in fields where true effect sizes are perceived to be small may be more likely to perform large studies than investigators working in fields where true effect sizes are perceived to be large. Or prejudice may prevail in a hot scientific field, further undermining the predictive value of its research findings. Highly prejudiced stakeholders may even create a barrier that aborts efforts at obtaining and disseminating opposing results. Conversely, the fact that a field is hot or has strong invested interests may sometimes promote larger studies and improved standards of research, enhancing the predictive value of its research findings. Or massive discovery-oriented testing may result in such a large yield of significant relationships that investigators have enough to report and search further and thus refrain from data dredging and manipulation.

## Most Research Findings Are False for Most Research Designs and for Most Fields

In the described framework, a PPV exceeding 50% is quite difficult to get. Table 4 provides the results of simulations using the formulas developed for the influence of power, ratio of true to non-true relationships, and bias, for various types of situations that may be characteristic of specific study designs and settings. A finding from a well-conducted, adequately powered randomized controlled trial starting with a 50% pre-study chance that the intervention is effective is eventually true about 85% of the time. A fairly similar performance is expected of a confirmatory meta-analysis of good-quality randomized trials: potential bias probably increases, but power and pre-test chances are higher compared to a single randomized trial. Conversely, a meta-analytic finding from inconclusive studies where pooling is used to “correct” the low power of single studies, is probably false if *R* ≤ 1:3. Research findings from underpowered, early-phase clinical trials would be true about one in four times, or even less frequently if bias is present. Epidemiological studies of an exploratory nature perform even worse, especially when underpowered, but even well-powered epidemiological studies may have only a one in five chance being true, if *R* = 1:10. Finally, in discovery-oriented research with massive testing, where tested relationships exceed true ones 1,000-fold (e.g., 30,000 genes tested, of which 30 may be the true culprits) [30,31], PPV for each claimed relationship is extremely low, even with considerable standardization of laboratory and statistical methods, outcomes, and reporting thereof to minimize bias.

**Table 4:**
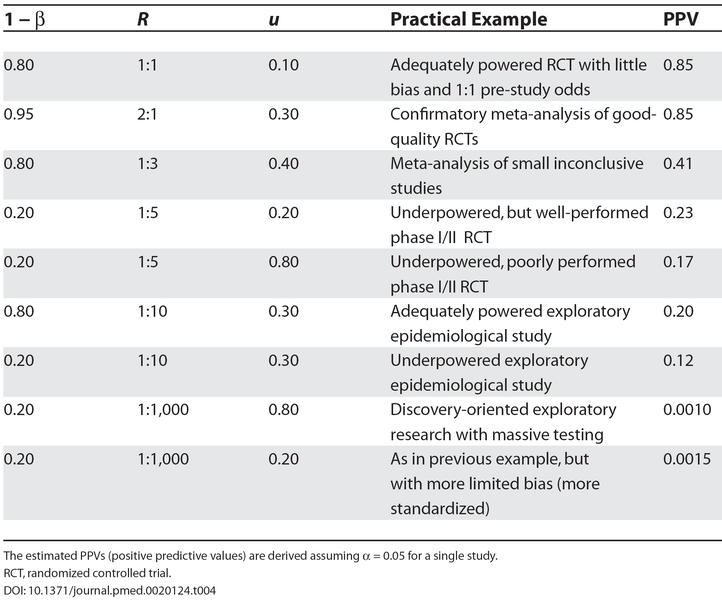
PPV of Research Findings for Various Combinations of Power (1 - ß), Ratio of True to Not-True Relationships *(R)*, and Bias *(u)*

The estimated PPVs (positive predictive values) are derived assuming a = 0.05 for a single study.

RCT, randomized controlled trial.

## Claimed Research Findings May Often Be Simply Accurate Measures of the Prevailing Bias

As shown, the majority of modern biomedical research is operating in areas with very low pre- and post-study probability for true findings. Let us suppose that in a research field there are no true findings at all to be discovered. History of science teaches us that scientific endeavor has often in the past wasted effort in fields with absolutely no yield of true scientific information, at least based on our current understanding. In such a “null field,” one would ideally expect all observed effect sizes to vary by chance around the null in the absence of bias. The extent that observed findings deviate from what is expected by chance alone would be simply a pure measure of the prevailing bias.

For example, let us suppose that no nutrients or dietary patterns are actually important determinants for the risk of developing a specific tumor. Let us also suppose that the scientific literature has examined 60 nutrients and claims all of them to be related to the risk of developing this tumor with relative risks in the range of 1.2 to 1.4 for the comparison of the upper to lower intake tertiles. Then the claimed effect sizes are simply measuring nothing else but the net bias that has been involved in the generation of this scientific literature. Claimed effect sizes are in fact the most accurate estimates of the net bias. It even follows that between “null fields,” the fields that claim stronger effects (often with accompanying claims of medical or public health importance) are simply those that have sustained the worst biases.

For fields with very low PPV, the few true relationships would not distort this overall picture much. Even if a few relationships are true, the shape of the distribution of the observed effects would still yield a clear measure of the biases involved in the field. This concept totally reverses the way we view scientific results. Traditionally, investigators have viewed large and highly significant effects with excitement, as signs of important discoveries. Too large and too highly significant effects may actually be more likely to be signs of large bias in most fields of modern research. They should lead investigators to careful critical thinking about what might have gone wrong with their data, analyses, and results.

Of course, investigators working in any field are likely to resist accepting that the whole field in which they have spent their careers is a “null field.” However, other lines of evidence, or advances in technology and experimentation, may lead eventually to the dismantling of a scientific field. Obtaining measures of the net bias in one field may also be useful for obtaining insight into what might be the range of bias operating in other fields where similar analytical methods, technologies, and conflicts may be operating.

## How Can We Improve the Situation?

Is it unavoidable that most research findings are false, or can we improve the situation? A major problem is that it is impossible to know with 100% certainty what the truth is in any research question. In this regard, the pure “gold” standard is unattainable. However, there are several approaches to improve the post-study probability.

Better powered evidence, e.g., large studies or low-bias meta-analyses, may help, as it comes closer to the unknown “gold” standard. However, large studies may still have biases and these should be acknowledged and avoided. Moreover, large-scale evidence is impossible to obtain for all of the millions and trillions of research questions posed in current research. Large-scale evidence should be targeted for research questions where the pre-study probability is already considerably high, so that a significant research finding will lead to a post-test probability that would be considered quite definitive. Large-scale evidence is also particularly indicated when it can test major concepts rather than narrow, specific questions. A negative finding can then refute not only a specific proposed claim, but a whole field or considerable portion thereof. Selecting the performance of large-scale studies based on narrow-minded criteria, such as the marketing promotion of a specific drug, is largely wasted research. Moreover, one should be cautious that extremely large studies may be more likely to find a formally statistical significant difference for a trivial effect that is not really meaningfully different from the null [32–34].

Second, most research questions are addressed by many teams, and it is misleading to emphasize the statistically significant findings of any single team. What matters is the totality of the evidence. Diminishing bias through enhanced research standards and curtailing of prejudices may also help. However, this may require a change in scientific mentality that might be difficult to achieve. In some research designs, efforts may also be more successful with upfront registration of studies, e.g., randomized trials [35]. Registration would pose a challenge for hypothesis-generating research. Some kind of registration or networking of data collections or investigators within fields may be more feasible than registration of each and every hypothesis-generating experiment. Regardless, even if we do not see a great deal of progress with registration of studies in other fields, the principles of developing and adhering to a protocol could be more widely borrowed from randomized controlled trials.

Finally, instead of chasing statistical significance, we should improve our understanding of the range of *R* values—the pre-study odds—where research efforts operate [10]. Before running an experiment, investigators should consider what they believe the chances are that they are testing a true rather than a non-true relationship. Speculated high *R* values may sometimes then be ascertained. As described above, whenever ethically acceptable, large studies with minimal bias should be performed on research findings that are considered relatively established, to see how often they are indeed confirmed. I suspect several established “classics” will fail the test [36].

Nevertheless, most new discoveries will continue to stem from hypothesis-generating research with low or very low pre-study odds. We should then acknowledge that statistical significance testing in the report of a single study gives only a partial picture, without knowing how much testing has been done outside the report and in the relevant field at large. Despite a large statistical literature for multiple testing corrections [37], usually it is impossible to decipher how much data dredging by the reporting authors or other research teams has preceded a reported research finding. Even if determining this were feasible, this would not inform us about the pre-study odds. Thus, it is unavoidable that one should make approximate assumptions on how many relationships are expected to be true among those probed across the relevant research fields and research designs. The wider field may yield some guidance for estimating this probability for the isolated research project. Experiences from biases detected in other neighboring fields would also be useful to draw upon. Even though these assumptions would be considerably subjective, they would still be very useful in interpreting research claims and putting them in context.

## Abbreviation

PPV: positive predictive value
