# Correction: Why Most Published Research Findings Are False

**DOI:** 10.1371/journal.pmed.1004085

**Published:** 2022-08-25

**Authors:** John P. A. Ioannidis

There is an error in Table 2. A set of parentheses is missing in the equation for Research Finding = Yes and True Relationship = No. Please see the correct Table 2 here.

**Table 2:** Research Findings and True Relationships in the Presence of Bias.

Research Finding	True Relationship		
	Yes	No	Total
Yes	(*c*[1 – β]*R + uc*β*R*)/(*R* + 1)	(*c*α + *uc*(1 – α))/*R* + 1)	*c*(*R* + α – β*R* + *u* – *uα* + *u*β*R*)/(*R +* 1)
No	(1 – *u*)*c*β*R*/(*R* + 1)	(1 – *u*)*c*(1 – α)/(*R* + 1)	*c*(1 – *u*)(1 – α + β*R*)/(*R* + 1)
Total	*cR*/(*R* + 1)	*c*/(*R* + 1)	*c*
